# Generation of Long Insert Pairs Using a Cre-LoxP Inverse PCR Approach

**DOI:** 10.1371/journal.pone.0029437

**Published:** 2012-01-09

**Authors:** Ze Peng, Zhiying Zhao, Nandita Nath, Jeff L. Froula, Alicia Clum, Tao Zhang, Jan-fang Cheng, Alex C. Copeland, Len A. Pennacchio, Feng Chen

**Affiliations:** Joint Genome Institute, United States Department of Energy, Walnut Creek, California, United States of America; UCLA-DOE Institute for Genomics and Proteomics, United States of America

## Abstract

Large insert mate pair reads have a major impact on the overall success of *de novo* assembly and the discovery of inherited and acquired structural variants. The positional information of mate pair reads generally improves genome assembly by resolving repeat elements and/or ordering contigs. Currently available methods for building such libraries have one or more of limitations, such as relatively small insert size; unable to distinguish the junction of two ends; and/or low throughput. We developed a new approach, **C**re-**L**oxP **I**nverse **P**CR **P**aired-**E**nd (CLIP-PE), which exploits the advantages of (1) Cre-LoxP recombination system to efficiently circularize large DNA fragments, (2) inverse PCR to enrich for the desired products that contain both ends of the large DNA fragments, and (3) the use of restriction enzymes to introduce a recognizable junction site between ligated fragment ends and to improve the self-ligation efficiency. We have successfully created CLIP-PE libraries up to 22 kb that are rich in informative read pairs and low in small fragment background. These libraries have demonstrated the ability to improve genome assemblies. The CLIP-PE methodology can be implemented with existing and future next-generation sequencing platforms.

## Introduction

*De novo* assembly of short reads generated by 2^nd^ generation sequencing platforms is a challenging task. Yet mate pair reads are useful for *de novo* assembly of complex genomes, especially for joining contigs flanking repetitive sequences. They can also be important for the discovery of structural variations, such as, insertions, deletions and inversions [1]. A variety of methods for constructing genomic DNA (gDNA) mate pair libraries have been developed for different sequencing platforms, each with its own pros and cons. Sanger paired end sequencing [2] generates long reads of high quality; however, the sequencing process is costly, labor intensive and time consuming. Genomic DNA di-tag is a method derived from SAGE (serial analysis of gene expression), in which 18 or 27 bp paired-end tags (PET) are extracted from the ends of gDNA inserts by *Mme*I [3] or *EcoP15*I [4] enzyme digestion. The resulting genomic fragments are concatenated to long fragments before being sequenced by Sanger or 2^nd^ generation platforms. The disadvantage of the di-tag method is that it produces short reads that may be mapped to multiple locations in complex genomes. An Illumina 40 kb jumping library was made by cloning 40 kb gDNA in a modified fosmid vector [5] and extracting paired end sequence tags via nick translation. However, the procedure is clone-based resulting in limited library complexity, and the vectors are not commercially available yet. Recently, various commercial kits became available for making mate pair libraries on 2^nd^ generation sequencing platforms. The Mate pair library prep kit (http://www.illumina.com/products/mate_pair_library_prep_kit.ilmn) offered by Illumina suggests constructing mate pair libraries not more than 5 kb in insert size; furthermore, because the junction of the two ends are not identifiable, the occurrence of chimeric reads containing part of two reads will increase significantly when sequencing read length increases. Utilizing the Cre-LoxP recombination system [6], [7], [8], the Roche 454 Jump Recombi Paired-end library preparation kit (http://www.454.com/products-solutions/experimental-design-options/multi-span-paired-end-reads.asp) makes up to 20 kb libraries. The advantage of this method is that longer reads can be obtained and there is a well defined junction site marked by the linker sequence that can be used to differentiate the origin of the reads with high confidence. However, their platform is not cost effective and the throughput is relatively low. More recently, Hillmer [9] reported a ligation-based approach (http://www3.appliedbiosystems.com/cms/groups/mcb_support/documents/generaldocuments/cms_081746.pdf, developed by Applied Biosystems SOLiD system) to create mate-paired library. The method uses either the nick translation or *EcoP15*I digestion to yield mate-paired genomic DNA. The junction site can be identified by the internal adaptor but the library insert size can be made up to 10 kb only.

**Figure 1:**
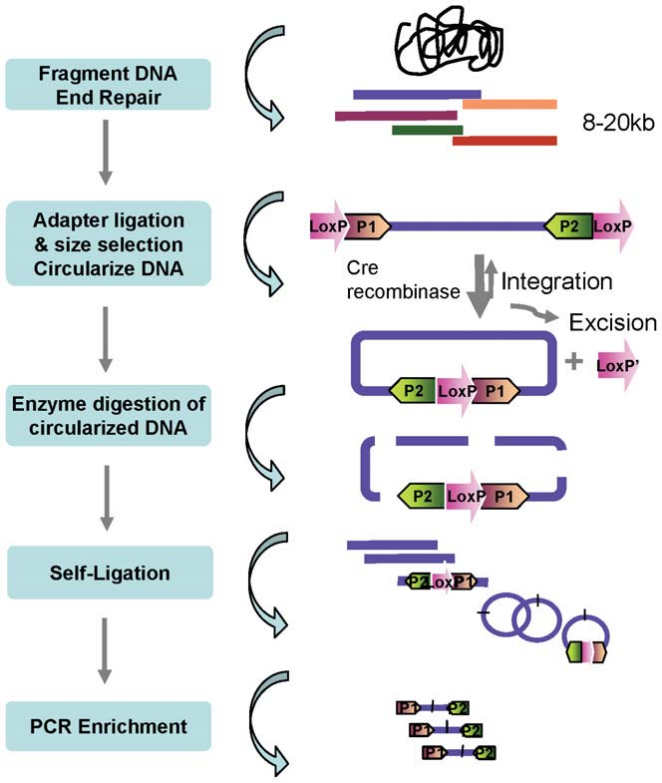
A schematic representation of the CLIP-PE library construction strategy. Following fragmentation, the DNA molecules are end-repaired and ligated with LoxP-P1 and LoxP-P2 adaptors integrated with Illumina P1 or P2 sequences. After separation and size selection, DNA is circularized by Cre recombinase, and non-circularized DNA is removed by exonuclease digestion. bp enzyme cutter is then used to digest and fragment DNA. (Alternatively, circularized DNA can be fragmented by random shearing to 400–500 bp followed by end-repair). DNA is then self-ligated. Inverse PCR with Illumina P1 and P2 PCR primers is used to enrich the mate paired molecules for sequencing. The final prepared libraries consist of short fragments made up of two DNA segments that were originally separately by 5–22 kb.

We report here a novel *in vitro* method that utilizes the Cre-LoxP recombination system and inverse PCR to make long insert mate-pair libraries. Briefly, randomly sheared gDNA fragment ends are ligated with adapters containing LoxP and Illumina P1 or P2 PCR priming sequences. Through Cre recombinase mediated intra-molecule recombination, gDNA fragments are circularized followed by enzymatic fragmentation and self ligation, DNA fragments containing P1-LoxP-P2 sequences are selectively amplified by PCR using Illumina P1 and P2 primers. The amplified products contain the paired end reads and are fully compatible with Illumina's sequencing platform. The CLIP-PE strategy is illustrated in Figure 1. This method has been used to generate 5 kb, 12 kb, and 22 kb Illumina mate pair libraries. Furthermore, a recognizable junction site has been introduced between read pairs to help demarcate them and to avoid chimeric reads.

## Results

### CLIP-PE libraries have a higher fraction of correctly distanced mate-pairs than Illumina jumping library

5 kb is the recommended insert size for Illumina's Mate pair kit. We created two 5 kb libraries of *Haloterrigena turkmenica VKM, DSM 551* using the CLIP-PE strategy and the Illumina's jumping method in parallel (Table 1). Both libraries were sequenced with the same 2×76 bps protocol using Illumina's Genome Analyzer (GA) IIx. Two criteria were used to measure the quality of a library: (1) the percentage of non-redundant pairs and (2) the percentage of chimeric pairs. Non-redundant pairs are those that have unambiguous mapping coordinates and are only counted once if they were duplicated. Small clonal artifact/contamination can easily be identified when a reference genome is supplied since this will map as read pairs closer to each other than was expected. Chimeric pairs are defined here as mapping to different chromosomes or in the wrong orientation.

**Table 1:** Results from the alignment of *Haloterrigena turkmenica VKM, DSM 5511* 5 kb mate pair libraries made with CLIP-PE method and Illumina Jumping method to the reference genome.

Description		CLIP		Jumping	
		Values	Percent	Values	Percent
Total reads		33,239,176		22,106,052	
Mapped paired reads		27,347,728		5,020,410	
Unambiguously mapped paired reads		27,028,366	98.8%	4,978,390	99.2%
Reads in non-redundant pair		6,329,048	23.1%	4,732,244	94.3%
Reads in non-redundant pair and >600 bp		5,642,986	20.6%	437,448	8.7%
Chimeric	Map to different chromosomes	538,004	2.0%	33,028	0.7%
	Wrong orientation	91,980	0.3%	427,056	8.5%
Number of gaps		7		767	
Mean gap size (bp)		8+/−13		41+/−133	

Percentages are calculated by dividing by “Mapped Paired Reads”. Non-redundant pairs map unambiguously to the reference and are de-replicated. The mean gap size and number of gaps are from the mate pair coverage, not raw read coverage.

**Figure 2:**
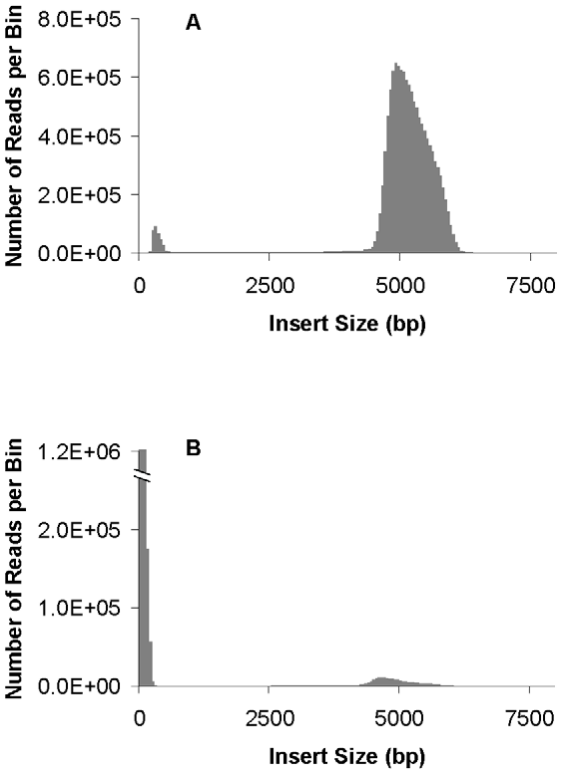
Histogram of insert lengths from the *Haloterrigena turkmenica VKM, DSM 5511* 5 kb mate pair libraries. A: CLIP-PE method, B: Illumina jumping method. The distribution of insert lengths was determined by aligning the reads to the reference genome.

From Table 1, we see that the CLIP-PE approach yielded 20.6% non-redundant pairs with the expected insert size (around 5 kb) compared to 8.7% from Illumina's jumping library. As with all the percentage calculations in this text, we will be dividing by the number of mapped paired reads and not the total number of reads since this will help normalize noise in the libraries like error rates and other variables affecting library quality. Figure 2 shows clearly that even though the Illumina's jumping library had a high percentage of uniquely mapped non-redundant pairs, most of them [91% = (4,732,244−437,448)/4,732,244] were derived from small fragments (<600 bp) whereas CLIP-PE had only 11% [(6,329,048−5,642,986)/6,329,048] that were too small. The higher percentage of good mate pairs in our CLIP-PE library is also reflected by higher clone coverage of the genome that can be defined as the average number of read pairs that span any given nucleotide in the reference. The average clone coverage of CLIP-PE versus Illumina's jumping library is 4,746× and 18×, respectively (Table S3). After mapping mate pair clones, the Illumina jumping library had 767 uncovered gaps with the average of 41+/−133 bp, while CLIP-PE library only has 7 gaps and 8+/−13 bp of mean gap size (Table 1). Lastly, the chimeric rate for CLIP-PE, at 2.3%, is better than the jumping method, 9.2%.

### CLIP-PE method can consistently generate high quality mate pair libraries

**Figure 3:**
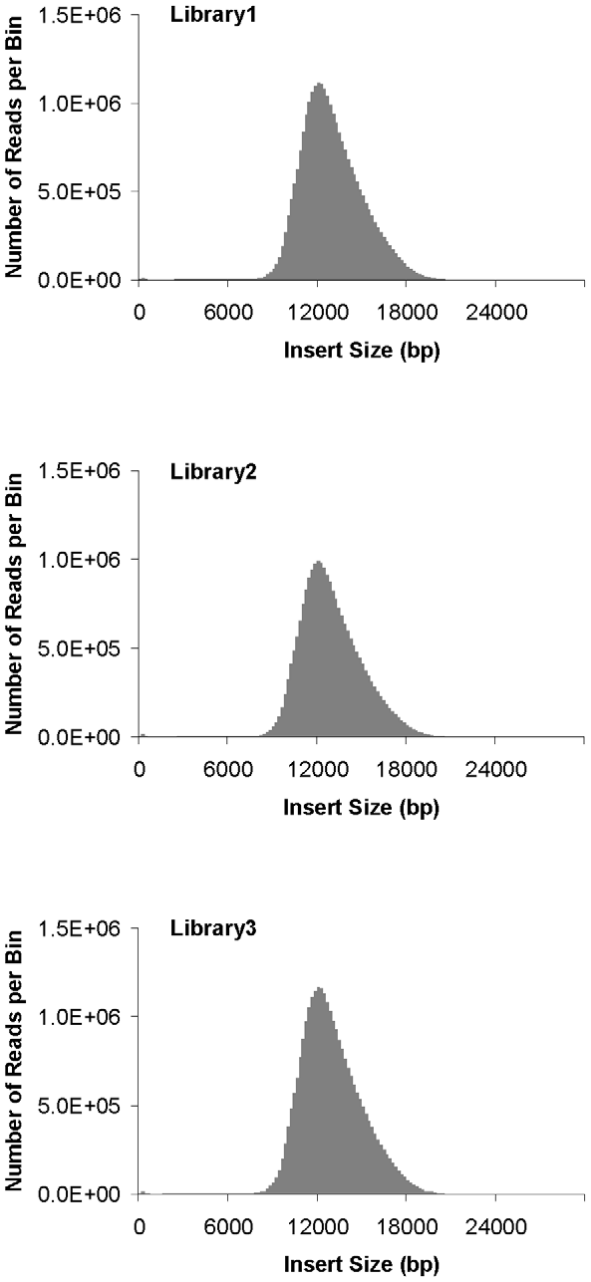
Histogram of insert sizes from *Saccharomyces cerevisiae* Illumina 12 kb CLIP-PE libraries. The distribution of insert lengths was determined by aligning the reads to the reference genome.

To test the CLIP-PE method with larger insert sizes, we made three *Saccharomyces cerevisiae* 12 kb libraries (Table 2 and Figure 3). Table 2 shows that the three 12 kb libraries were of high quality and highly reproducible (see Table S4 for more detailed results). For instance, averages of 59% of the mapped paired reads were unique non-redundant pairs with the expected insert size. Roughly 5–7% of total reads mapped to different chromosomes and less than 0.05% mapped in the wrong orientation. We also successfully created three 22 kb libraries from *S. cerevisiae* genomic DNA (Table 3) and these will be discussed more in the next session. *S. cerevisiae* is much more repetitive than *H. turkmenia*, thereby affecting the rates of unambiguously mapped reads. We assume that this accounts for the difference seen.

**Table 2:** Results from the alignment of *Saccharomyces cerevisiae* Illumina 12 kb CLIP-PE libraries to the reference genome.

Description		Library 1		Library 2		Library3	
		Values	Percent	Values	Percent	Values	Percent
Total reads		74,789,134		67,341,574		79,458,906	
Mapped paired reads		67,120,758		59,335,508		69,864,792	
Unambiguously mapped paired reads		50,784,982	75.7%	44,680,512	75.3%	53,095,212	76.0%
Reads in non-redundant pair		39,696,694	59.1%	34,717,654	58.5%	41,471,704	59.4%
Reads in non-redundant pair and >600 bp		39,666,120	59.1%	34,662,488	58.4%	41,436,998	59.3%
Chimeric	Map to different chromosomes	3,627,494	5.4%	3,999,848	6.7%	4,553,416	6.5%
	Wrong orientation	20,680	0.0%	22,050	0.0%	27,360	0.0%
Number of gaps		26		26		24	
Mean gap size (bp)		84+/−157		299+/−1268		65+/−112	

Percentages are calculated by dividing by “Mapped Paired Reads”. Non-redundant pairs map unambiguously to the reference and are de-replicated. The mean gap size and number of gaps are from the mate pair coverage, not raw read coverage.

**Table 3:** Results from the alignment of *Saccharomyces cerevisiae* Illumina 22 kb CLIP-PE libraries to a reference genome.

Description		NlaIII cut(4 bp overhang)		HpyCH4IV cut(2 bp overhang)		Random shearing(blunt end)	
		Values	Percent	Values	Percent	Values	Percent
Total reads		45,036,914		38,287,456		41,663,612	
Mapped paired reads		40,760,246		24,523,852		30,241,684	
Unambiguously mapped paired reads		31,789,650	78.0%	19,898,350	81.1%	24,834,322	82.1%
Reads in non-redundant pair		4,573,952	11.2%	1,076,742	4.4%	1,545,550	5.1%
Reads in non-redundant pair and >600 bp		4,530,782	11.1%	974,198	4.0%	744,022	2.5%
Chimeric	Map to different chromosomes	697,566	1.7%	361,526	1.5%	473,038	1.6%
	Wrong orientation	6,250	0.0%	4,658	0.0%	5,684	0.0%
Number of gaps		27		27		25	
Mean gap size (bp)		137+/−285		179+/−499		108+/−194	

Percentages are calculated by dividing by “Mapped Paired Reads”. Non-redundant pairs map unambiguously to the reference and are de-replicated. The mean gap size and number of gaps are from the mate pair coverage, not raw read coverage.

### Ligation efficiency affects the productivity and quality of CLIP-PE libraries

**Figure 4:**
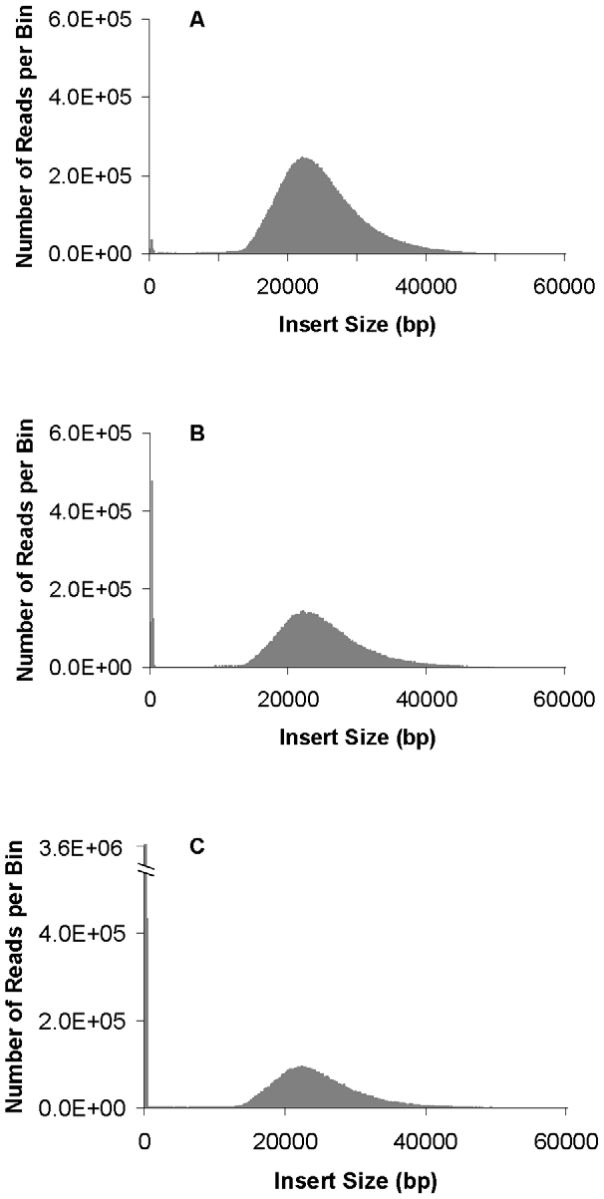
Histogram of insert sizes from *Saccharomyces cerevisiae* Illumina 22 kb CLIP-PE libraries. A: cut with NlaIII, B: cut with HpyCh4IV, C: random shearing approach. The distribution of insert lengths was determined by aligning the reads to the reference genome.

During the CLIP-PE process, either random shearing or frequent restriction enzyme cutting can be used for the secondary fragmentation after Cre circularization. To see the effects on ligation efficiency, we compared the two methods of fragmentation during the creation of three 22 kb *S. cerevisiae* CLIP-PE libraries. Restriction digestion was used for two libraries and random shearing for the third. Only 4 base cutting enzymes that had no cutting site in the P1-LoxP-P2 fragment were used. Two different 4 bp restriction enzymes were selected, from which, NlaIII generated 4 bp overhangs and HpyCH4IV generated 2 bp overhangs. Judging by the proportion of non-redundant pairs, the NlaIII library was the most efficient (11.1% non-redundant) followed by the 2 bp overhang, HpyCH4IV library (4.0%) and finally the blunt end, randomly sheared library (2.5%) (Table 3). This result is expected since self-ligation with 4 bp overhang is more efficient than 2 bp overhang that is more efficient than blunt end. Figure 4 clearly shows that the proportion of read pairs with short insert sizes increases as the size of the overhang gets smaller. All libraries had low (∼1.5–1.7%) chimeric pairs (Table 3) and almost no gaps in clone coverage (Table S5).

### Genome assemblies are significantly improved by combining short Illumina reads with long paired end reads generated by the CLIP-PE method

**Figure 5:**
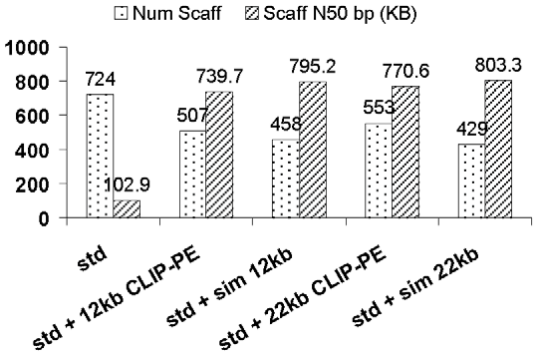
Assembly metrics for *Saccharomyces cerevisiae* Illumina CLIP-PE libraries. *std* refers to standard Illumina 250 bp library, *sim 12 kb* refers to simulated 12 kb mate pair library, and *sim 22 kb* refers to simulated 22 kb mate pair library.

By combining standard Illumina short insert reads in addition to large insert mate-pair reads, repetitive regions can be resolved during assembly. We tested if 12 kb or 22 kb *S. cerevisiae* CLIP-PE libraries, after normalization by 10 million equal numbers of reads, helped with the genome assembly when combined with short insert (250 bp) 2×76 bp data. In addition to our 12 and 22 kb CLIP-PE libraries, we made simulated mate pair libraries of the same insert lengths as a comparison. The combinations used for assembly were: (1) standard Illumina short 250 bp library alone; (2) standard short plus 12 kb CLIP-PE library; and (3) standard short plus 22 kb CLIP-PE library; (4) the simulated 12 kb and 22 kb libraries in place of our CLIP-PE libraries. Four criteria were used to assess final assemblies: (1) number of bases assembled; (2) the N50 scaffold and contig size; (3) the number of scaffolds and contigs (Figure 5 and Table S6); and (4) the number of mis-assemblies (Table 4). The results did not show a significant difference in the number of assembled bases for any given assembly (11.6–12.1 MB), which is near the expected genome size of *S. cerevisiae* (12.2 MB). However, assemblies using CLIP-PE libraries greatly improved scaffold size when compared to the standard dataset alone. For example, the standard only assembly had an N50 scaffold size of 102.9 kb whereas the hybrid assemblies using our CLIP-PE libraries had scaffold N50 values of 739.7 kb (for 12 kb library) and 770.6 kb (for 22 kb library). This 7-fold jump in the N50 value is comparable to the scaffold N50 of the simulated hybrid assemblies. The number of scaffolds decreases from 724 to 507 in the CLIP-PE 12 kb+standard assembly and 724 to 553 for the CLIP-PE 22 kb+standard assembly. We did not observe that 22 kb mate pair library performs better than 12 kb library in assembly. There could be two reasons: in comparison of assembly of simulated data, 22 kb and 12 kb mate pairs performed almost identically; and in addition to this, 22 kb mate pair library is more difficult to construct and may have lower coverage and/or lower complexity. Values for the number of mis-assemblies including relocations, translocation, and inversions are reasonably similar (Table 4). Overall, results are comparable between contigs assembled using CLIP-PE or the simulated mate-pair reads, suggesting that CLIP-PE library quality is very high. Our CLIP-PE libraries of other microbes have consistently shown to help genome assembly and finishing (data not shown).

**Table 4:** Mis-assembly numbers of *Saccharomyces cerevisiae* CLIP-PE libraries.

Assembly Library Type	Relocations	Translocations	Inversions
std+12 kb_CLIP-PE	29	10	10
std+sim 12 kb	31	8	0
std+22 kb_CLIP-PE	46	7	0
std+sim 22 kb	52	7	2

*Std* refers to standard Illumina 250 bp library, *sim 12 kb* refers to simulated 12 kb mate pair library, *sim 22 kb* refers to simulated 22 kb mate pair library.

## Discussion

Next-generation sequencing technologies produce huge amounts of data but the short read length (∼100 bp as compared to the ∼700 bp in the capillary method) presents a problem when trying to assemble the reads, especially of long repeat and duplicated regions of the genome. To overcome these problems, *de novo* genome assemblies require large insert, mate pair libraries. Since the Cre-LoxP system can circularize greater than 90 kb DNA fragments with high efficiency [10], we employ the Cre-LoxP recombination rather than ligation used in Illumina jumping method to circularize gDNA fragments. Although some larger insert libraries such as fosmid, PAC or BAC can generate large insert paired ends, they are all constructed *in vivo*, which is clone based, therefore resulting in limited library complexity. In our experience, libraries made through Cre-loxP system not only produce more paired-end reads than ligation based method, but also have potential to make larger (>20 kb) insert size mate pair libraries to replace those *in vivo* methods.

5 kb *H. turkmenica* CLIP-PE libraries has fewer percentage of reads in non-redundant pairs than *S. cerevisiae*'s 12 kb libraries, 23.1% versus 59%. These are two non-parallel experiments carried out in two different times. 5 kb H. *turkmenica* library was constructed by using less Cre-loxP reactions (one versus four for 12 kb *S. cerevisiae* library). In addition, the 5 kb *H. turkmenica* CLIP-PE library was generated prior to the optimization of Cre recombination and the inverse PCR steps that were implemented in *S. cerevisiae*'s CLIP-PE libraries. Most likely, these reasons contribute to the high redundancy of 5 kb *H. turkmenica* CLIP-PE library. It has to be noticed that as the size of DNA molecule increases, the recombination efficiency of Cre-LoxP system seems decreased. This is probably one of the reasons causing the low complexity of 22 kb library comparing to the 12 kb libraries, (11.2% of reads in non-redundant pairs versus ∼59%), not neglecting less input molecules of larger fragment with same mass of DNA in circularization step.

Utilizing an inverse PCR strategy in our CLIP-PE procedure brings us several benefits. Current paired-end library generation method from 454 involves two ligation steps, where linkers and sequencing adapters are separately ligated after the first and second fragmentation of DNA. For both ligation steps, only molecules with the correct combination of linkers and adapters will result in useful products. Because identical adapters are ligated to both ends, Applied Biosystems SOLiD mate-paired library generation method does not require correct DNA molecule and adapter combination in the ligation step after the first fragmentation. However, circularization in the method is mediated through an internal linker and the efficiency of circularization is relatively low. Additionally, their process includes a total of three ligation steps and the last ligation step still requires correct combination of ends and adapters. By integrating Illumina amplification adapter P1 and P2 with LoxP sequence (Figure 1), our CLIP-PE method requires correct DNA molecule and adapter combination in only one ligation step resulting in a higher yield and complexity of the final library. This strategy also simplifies the procedure when restriction digestion approach is used for second fragmentation, since no end repair of the DNA molecule is necessary for self-ligation. Only the recombined DNA fragments with P1-LoxP-P2 structure can be amplified through inverse PCR after self-ligation. The CLIP-PE strategy provides an efficient way to enrich the desired DNA fragments with two ends of original large DNA molecules brought together by recombination.

The Illumina mate pair library protocol utilizes self-ligation to bring two ends of a large DNA fragment together without a linker or recognizable sequence pattern. After sequencing, the junction point of the two ends cannot be identified. Thus, Illumina recommends the sequencing read length as short as 36 bp to prevent the risk of reading through the junction site which will result in chimeric reads. Our CLIP-PE procedure allows us to identify the junction site since the site is the same as the restriction site of the enzyme used. By trimming reads after first restriction site, chimeric reads can be avoided. This makes sequencing longer reads (2×76 bp or more) possible and will greatly aid in downstream data analysis and assembly. Alternatively, linkers can be used to identify junction sites as in 454 and SOLiD library generation methods. Our results indicated that this approach was not as effective as using enzyme cutting method, probably due to low ligation efficiency (data not shown). Additionally, since ends with 4 bp overhangs have higher ligation efficiency than the blunt ends, we get more than four-fold (11.1%/2.5%) increase in non-redundant mate pair reads and 26-fold [(5.1%–2.5%)/(11.2%–11.1%)] less non-specific background (i.e.: fragments less than 600 bp).

Because enzyme-cutting sites may not be evenly distributed throughout the genome, there may be concerns about potential gaps in genome coverage when using a restriction enzyme in the second fragmentation step. Our method randomly shears the genomic DNA in the first fragmentation step and the potential non-randomness of the restriction digestion in the second fragmentation step will be compensated for by the depth of randomly sheared fragments. In rare cases, where restriction enzyme cutting sites are very unevenly distributed, for example, in extreme high or low GC genomes, combining reads from libraries of two or more enzymes would most likely eliminate such coverage bias. There are many 4 bp enzymes available for the CLIP-PE second fragmentation step (Table S2). So far, even with one enzyme (NlaIII), we did not detect any bias in clone representation for six genomes with variable GC content ranging from 28% to 74% (data not shown).

The unique features of the large mate pair libraries created by the CLIP-PE method deliver unmatched benefits compared to the Illumina jumping method. It generates a higher number of mate-paired reads with the desired insert size and known junction site. In addition to the libraries we made *in vitro* with various insert sizes, we applied the CLIP-PE strategy to *in vivo* systems such as fosmid cloning to achieve larger and tightly controlled insert size (paper in preparation). Combined with a standard shotgun library, CLIP-PE will streamline *de novo* genome assembly and the finishing process. It also has prospects for genomic analysis such as structural variation detection especially for large complex genomes [11]. Furthermore, the CLIP-PE strategy is versatile and can be widely applicable to other next-generation sequencing platforms.

## Materials and Methods

### Illumina library preparation

Illumina standard shotgun libraries were created with commercial Illumina Pair-end kit using 1 ug of genomic DNA without PCR amplification. Illumina jumping libraries were created with commercial Illumina's Mate-pair library preparation kit V2 with 5 ug genomic DNA.

### CLIP-PE library preparation

CLIP-PE libraries were prepared as follows: (i) 5, 15 or 30 ug of genomic DNA in 150 ul of EB buffer was sheared (Genomic Solutions, HydroShear) to a desired size: 5 kb, 12 kb, or 22 kb, respectively; (ii) 5 ul each of T4 DNA polymerase (New England Biolabs (NEB) M0203), Klenow enzyme (NEB, M0210L) and T4 Polynucleotide Kinase (NEB, M0201) and dNTP (NEB, N0447L) (400 uM final), BSA (0.1 ug/ul final) were used to repair the ends in 200 ul volume of 1× TNK buffer for 20 minutes at 25°C; (iii) after end repair, 1.5 volume of Genfind v2 beads (Agencourt, A41499) were used to purify DNA according the manufacturer's guide, DNA was eluted with 40 ul EB; (iv) 2.5 ul of 20 uM each LoxP-P1 and LoxP-P2 integrated adapters (Table S1) were ligated to the ends of DNA with 5 ul/100 ul of Quick ligase (NEB, M2200) for 15 minutes at 25°C; (v) for 5 and 12 kb library, adapter ligated DNA was size selected through regular gel electrophoresis [1×TAE, 0.8% Ultrapure agarose (Invitrogen, 16500100), 0.6 v/cm, overnight] and purified with Wizard® SV Gel and PCR Clean-Up System (Promega, A9281); for 22 kb library, the DNA was size selected with Pulse-Field gel electrophoresis (PFGE, 0.5× TBE, 1% Ultrapure agarose, 6 v/cm, 120°, 0.1–7 s pulse, 14°C, 11 hrs), DNA fragment was cut with out dye staining and electro-eluted (6 V/cm, 90 min, reverse current 20 seconds) in dialysis bags (Sigma-Alorich, D0405) and concentrated to 40 ul volume by YM-100 columns (Millipore, 42412,) by 500×g centrifugation, dilute with 250 ul of EB and concentrated to 40 ul volume again; (vi) DNA was filled-in with 24 u of *Bst* DNA polymerase (NEB, M0275) and dNTP (800 uM final) in 50 ul volume for 15 minutes at 50°C and quantified by Qubit dsDNA BR kit (Invitrogen, Q32850); (vii)1–4 of LoxP-Cre reactions (300 ng DNA/2–10 u Cre-recombinase/100 ul) were set up for 45 minutes at 37°C; then 10 minutes at 70°C; linear DNA was digested away by adding ATP (1 mM final) and 2 u/100 ul of Plasmid-Safe™ ATP-Dependent DNase (Epicentre, E3101 K) and incubate 30 minutes at 37°C then 30 minutes at 70°C, followed by EtOH precipitation purification; (viii) the circularized DNA was digested by 10 u/50 ul of NlaIII (NEB, R0125) for 1–2 hour at 37°C and heat inactivation 20 minutes at 65°C; (ix) ATP, T4 ligase buffer and T4 ligase (NEB, M0202) were added directly to the digestion reaction (adjust DNA concentration to 1 ng/ul, ATP 1 mM final, 1 ul T4 ligase/20 ul volume) to self-ligate of DNA fragments at room temperature for 1 hr or 14°C overnight; (x) Optional: add Plasmid-Safe™ ATP-Dependent DNase (1 u/100 ul) directly to the self ligation solution to digest away linear DNA (xi) the ligation product was purified by EtOH precipitation or Streptavidin beads (Invitrogen Dynabeads® M-270 Streptavidin) according to the manufacturer's guide (xii) inverse PCR with Illumina pair-end library primers (Table S1) and Phusion DNA Polymerase (NEB) were used to amplify the molecules containing the mate-pair ends only. (xiii) The PCR products were purified with gel electrophoresis (1× TAE, 1.5% agarose 5 V/cm, 60 minutes). Gel piece containing 300–600 bp DNA fragments was extracted using a Wizard SV column.

### Illumina sequencing

Sequencing was carried out according to the manufacturer's recommended protocols on a Genome analyzer IIx (GAIIx). For standard Illumina PE libraries, a sequencing run was 2×100 cycles and data was trimmed to 76 bp based on average quality scores for assembly analysis. All other sequencing runs were performed at 2×76 cycles.

### Post-sequencing analysis

To reduce the probability of a read crossing the junction point where the two distant ends of the original DNA fragment were joined during circularization, Illumina recommends reads no longer than 36 nucleotides when sequencing mate-pair libraries. Thus, we trimmed the sequencing results from Illumina jumping libraries to 35 bp. For CLIP-PE libraries, we trimmed bases after the restriction enzyme recognition site. The average read length is 70 bp after trimming and 23% of the reads containing restriction enzyme cutting site. All reads were aligned to the reference using the BWA aligner [12]. Fast and accurate short read alignment with Burrows-Wheeler transform [13].

### Data simulations and genome assembly

Simulated reads were generated from the reference using wgsim version 0.2.3 [12] with a read length of 76 bp and an error rate of 1%. Datasets were assembled with velvet [13]. Various hash lengths (kmer lengths) were tested depending on the read length as well as varying the minimum number of pairs required to make a join. Libraries were specified as short pairs and an approximate insert size was given to velvet. Auto settings were used for the coverage cutoff and expected coverage variables. A minimum contig length of 200 bp was specified. Assembly accuracy was evaluated using dnadiff (http://www.gnu-darwin.org/www001/ports-1.5a-CURRENT/biology/mummer/work/MUMmer3.20/docs/dnadiff.README) [14] to compare the assembly to the reference. Relocations are defined by dnadiff as “number of breaks in the alignment where adjacent 1-to-1 blocks are in the same sequence but not consistently ordered”. Translocations are where adjacent blocks are in different sequences and inversions are when the blocks are inverted.

## Supporting Information

Table S1 **Sequences of CLIP-PE adapters and PCR primers.** All oligonucleotides were purchased from IDT with HPLC purification (www.idtdna.com). T*: biotin labeled Thymine (optional). Adaptor annealing method: 1) dissolve each primers with TE0.1 buffer, 2) mix 10 ul of top and 10 ul of bottom primer with 30 ul of TE0.1 that contains 50 mM NaCl, 3) anneal primers in a thermocycler using following program: 95°C for 1 minute; decrease temperature 0.1°C/second to 15°C final temperature; 4°C forever. CLIP-PE PCR primers: oligonucleotide sequences for © 2007–2011 Illumina, Inc. All rights reserved. Derivative works created by Illumina customers are authorized for use with Illumina instruments and products only. All other uses are strictly prohibited.
(PPT)

Table S2 **Candidates of 4 bp restriction enzymes used for CLIP-PE.**
(PPT)

Table S3 **Detailed data of comparison of CLIP-PE with jumping method.**
(PPT)

Table S4 **Detailed data of three** ***Saccharomyces cerevisiae*** **12 kb CLIP-PE libraries.**
(PPT)

Table S5 **Detailed data of** ***Saccharomyces cerevisiae*** **22 kb CLIP-PE libraries made by enzyme cutting and random shearing.**
(PPT)

Table S6 **Detailed assembly metrics using combinations of real and simulated CLIP-PE libraries from** ***Saccharomyces cerevisiae*** **.** The data sets have been normalized with equal number (10 million) of reads. Sim: simulated; Std: standard; Scaff: scaffold; Ctg: contig; Num: number.
(PPT)
