# Supplementary figures and images for: Generation of Long Insert Pairs Using a Cre-LoxP Inverse PCR Approach
Source: PLoS One. 2012 Jan 9;7(1):e29437. doi: 10.1371/journal.pone.0029437 (PMC3253782; doi:10.1371/journal.pone.0029437)

## Slide 1
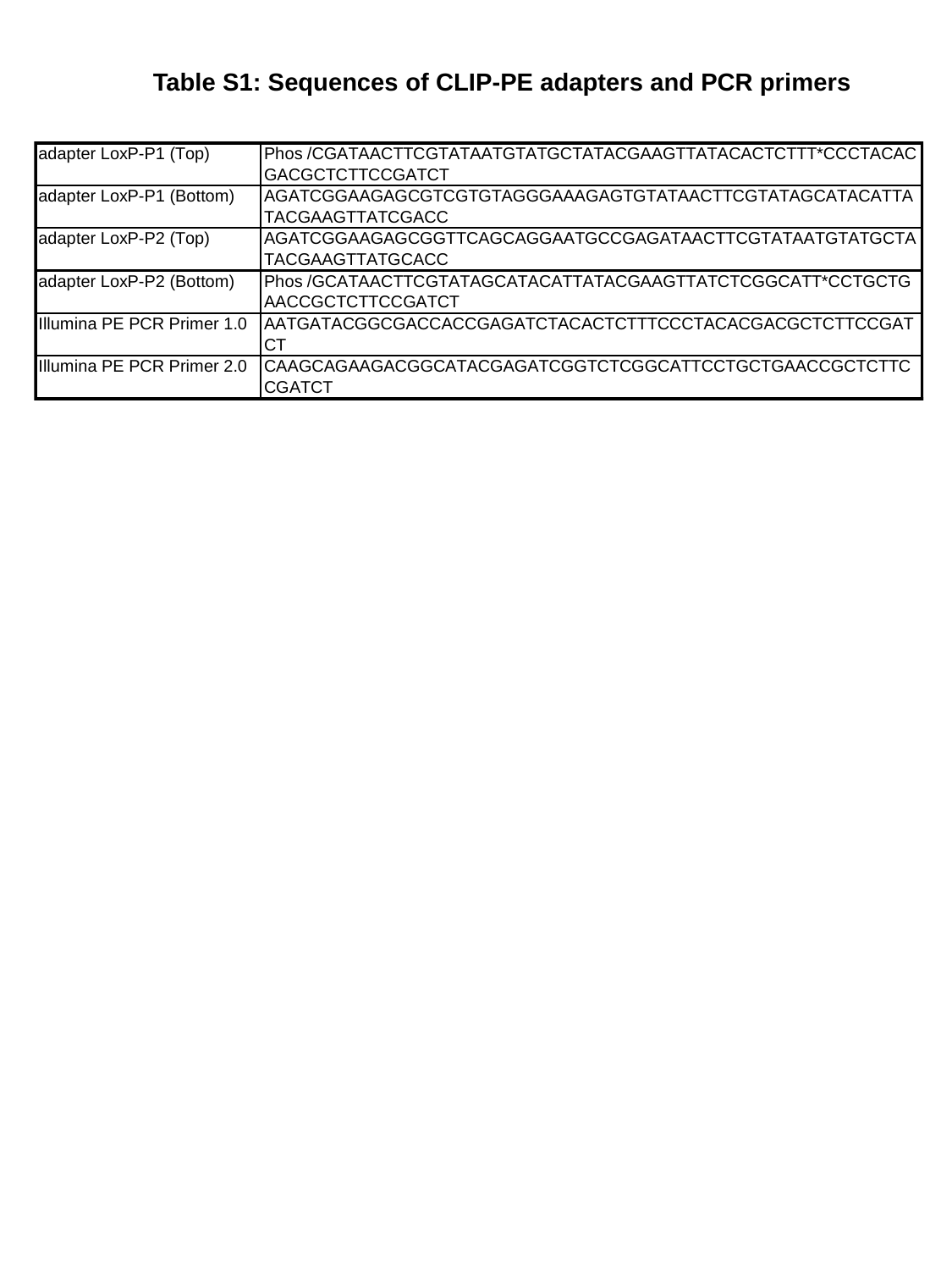

Table S1: Sequences of CLIP-PE adapters and PCR primers

## Slide 2
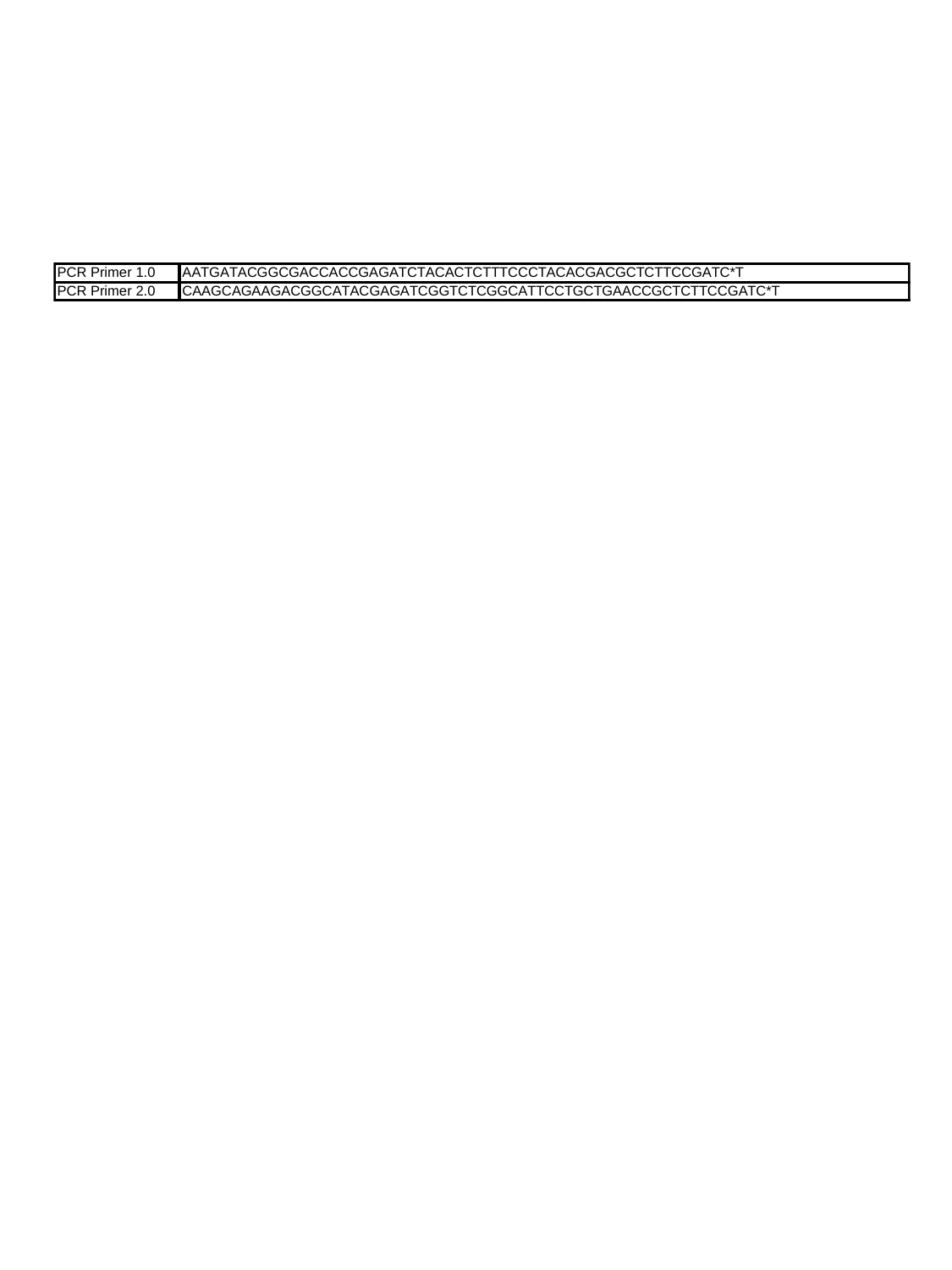

Supplement: Table S1 — Sequences of CLIP-PE adapters and PCR primers. All oligonucleotides were purchased from IDT with HPLC purification (www.idtdna.com). T*: biotin labeled Thymine (optional). Adaptor annealing method: 1) dissolve each primers with TE0.1 buffer, 2) mix 10 ul of top and 10 ul of bottom primer with 30 ul of TE0.1 that contains 50 mM NaCl, 3) anneal primers in a thermocycler using following program: 95°C for 1 minute; decrease temperature 0.1°C/second to 15°C final temperature; 4°C forever. CLIP-PE PCR primers: oligonucleotide sequences for © 2007–2011 Illumina, Inc. All rights reserved. Derivative works created by Illumina customers are authorized for use with Illumina instruments and products only. All other uses are strictly prohibited. (PPT) [file pone.0029437.s001.ppt]

## Slide 1
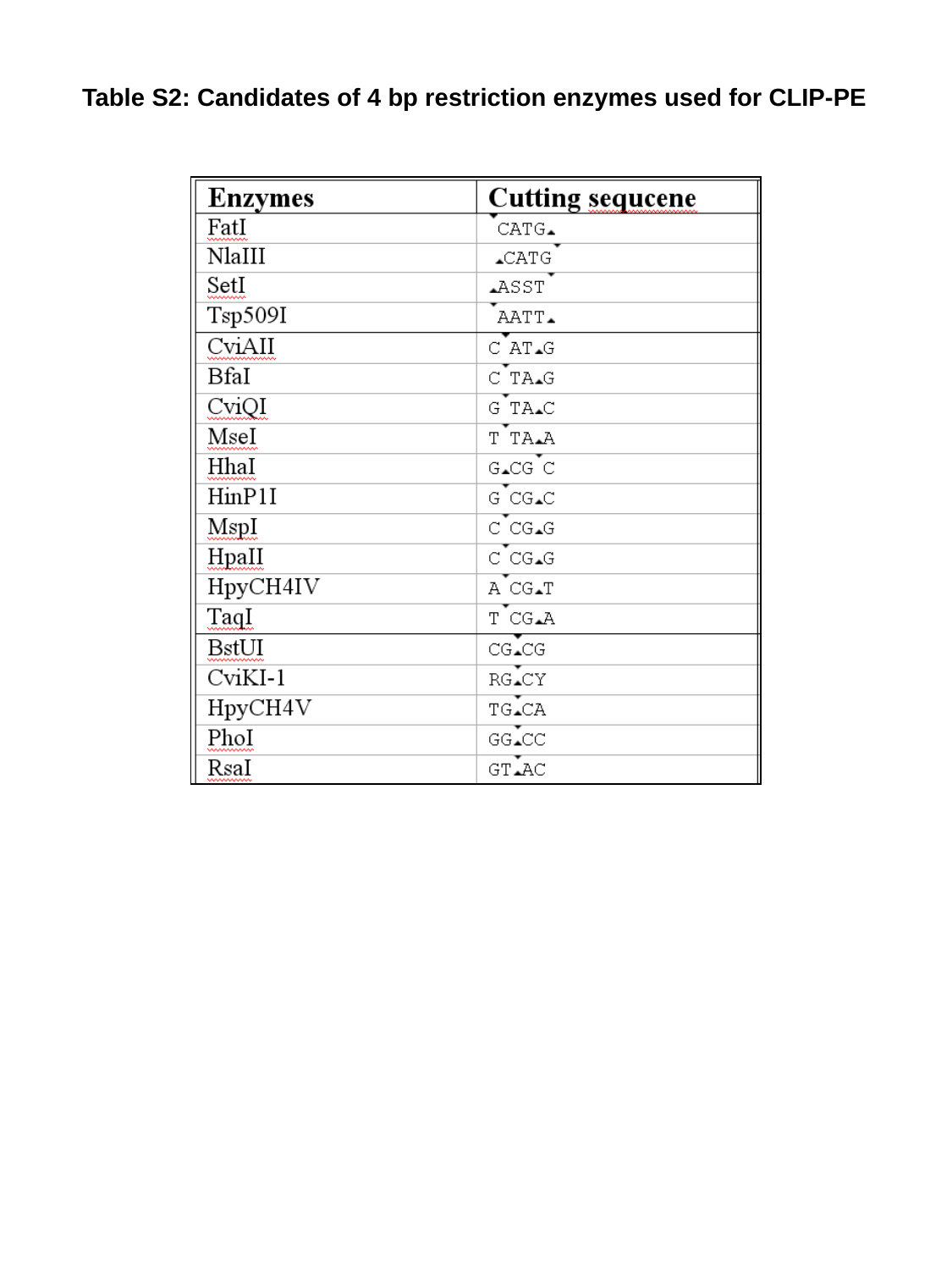

# Table S2: Candidates of 4 bp restriction enzymes used for CLIP-PE

Supplement: Table S2 — Candidates of 4 bp restriction enzymes used for CLIP-PE. (PPT) [file pone.0029437.s002.ppt]

## Slide 1
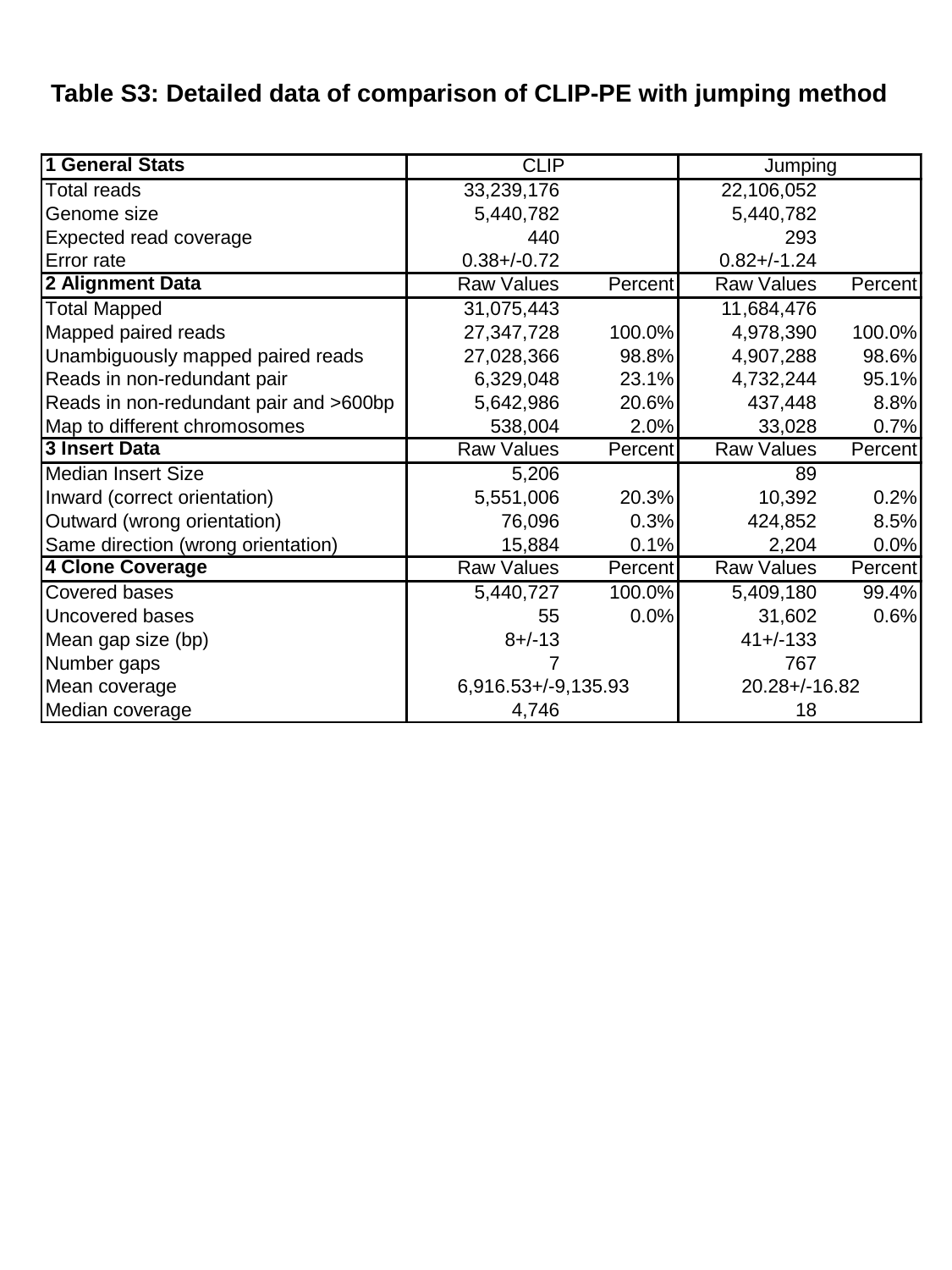

Table S3: Detailed data of comparison of CLIP-PE with jumping method

Supplement: Table S3 — Detailed data of comparison of CLIP-PE with jumping method. (PPT) [file pone.0029437.s003.ppt]

## Slide 1
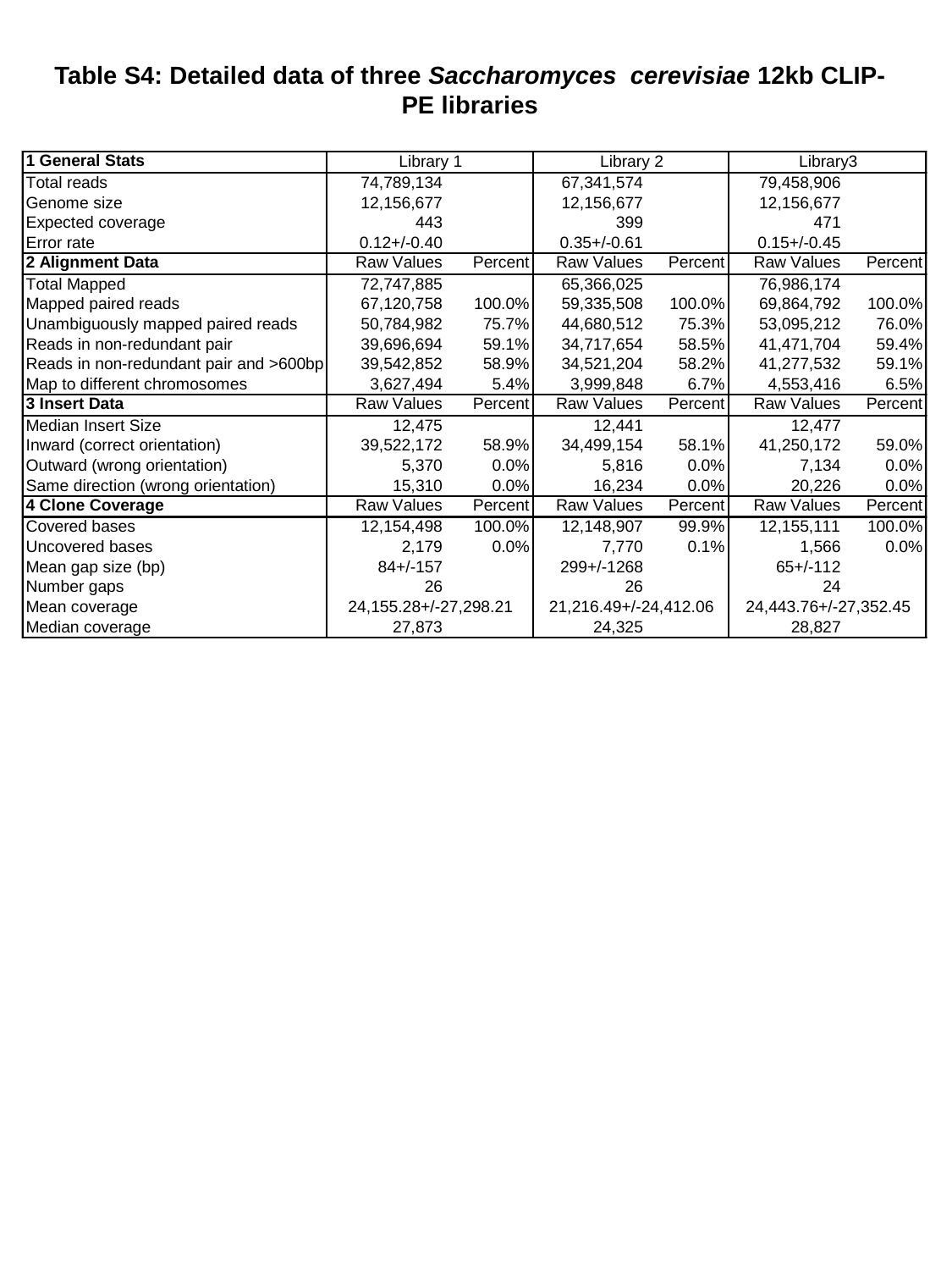

Table S4: Detailed data of three Saccharomyces cerevisiae 12kb CLIP-PE libraries

Supplement: Table S4 — Detailed data of three Saccharomyces cerevisiae 12 kb CLIP-PE libraries. (PPT) [file pone.0029437.s004.ppt]
