# Supplementary material for: Generation of Long Insert Pairs Using a Cre-LoxP Inverse PCR Approach
Source: PLoS One. 2012 Jan 9;7(1):e29437. doi: 10.1371/journal.pone.0029437 (PMC3253782; doi:10.1371/journal.pone.0029437)
Supplement: Table S5 — Detailed data of Saccharomyces cerevisiae 22 kb CLIP-PE libraries made by enzyme cutting and random shearing. (PPT) [file pone.0029437.s005.ppt]

## Slide 1
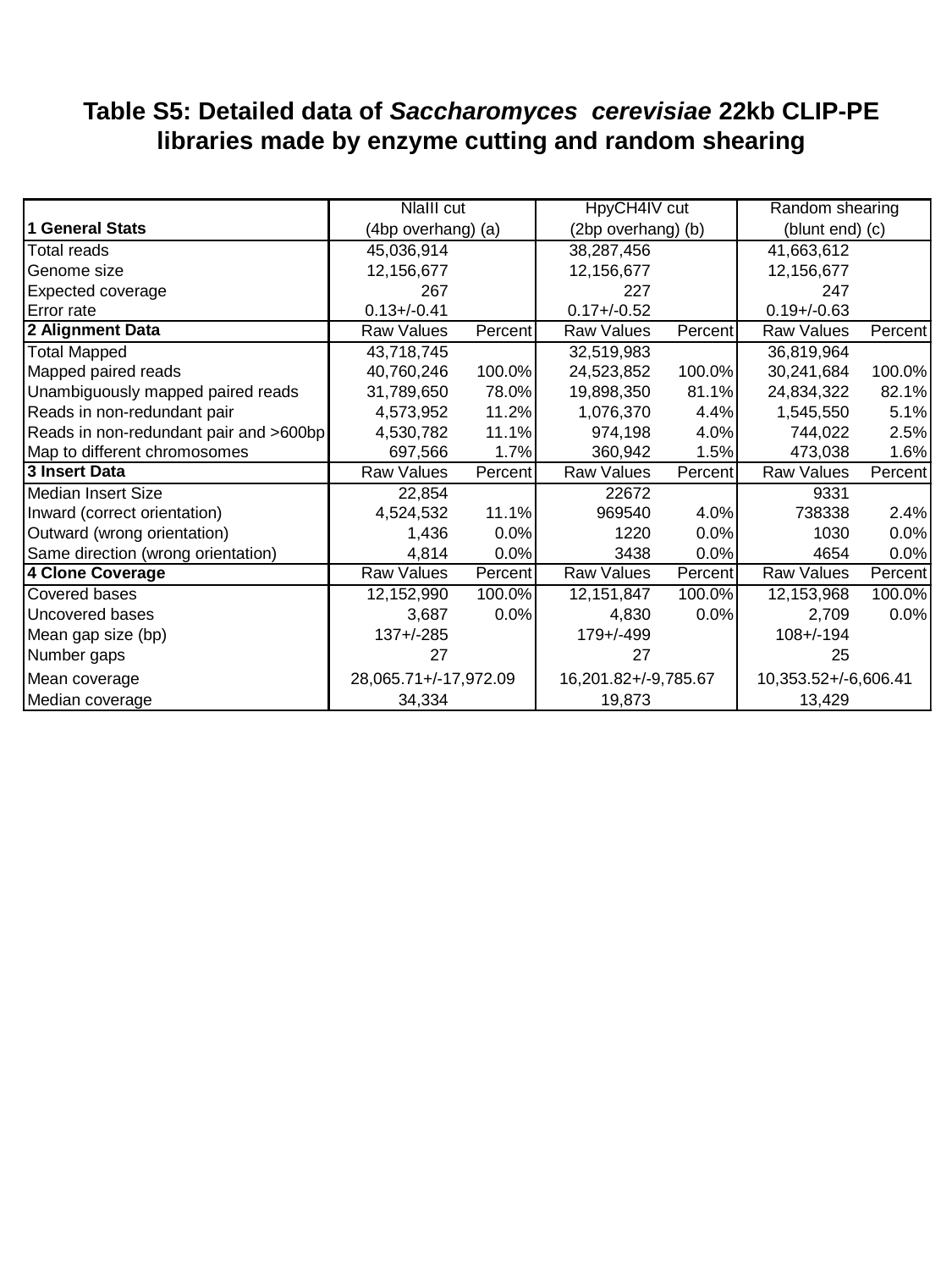

Table S5: Detailed data of Saccharomyces cerevisiae 22kb CLIP-PE libraries made by enzyme cutting and random shearing
