# Supplementary material for: Generation of Long Insert Pairs Using a Cre-LoxP Inverse PCR Approach
Source: PLoS One. 2012 Jan 9;7(1):e29437. doi: 10.1371/journal.pone.0029437 (PMC3253782; doi:10.1371/journal.pone.0029437)
Supplement: Table S6 — Detailed assembly metrics using combinations of real and simulated CLIP-PE libraries from Saccharomyces cerevisiae . The data sets have been normalized with equal number (10 million) of reads. Sim: simulated; Std: standard; Scaff: scaffold; Ctg: contig; Num: number. (PPT) [file pone.0029437.s006.ppt]

## Slide 1
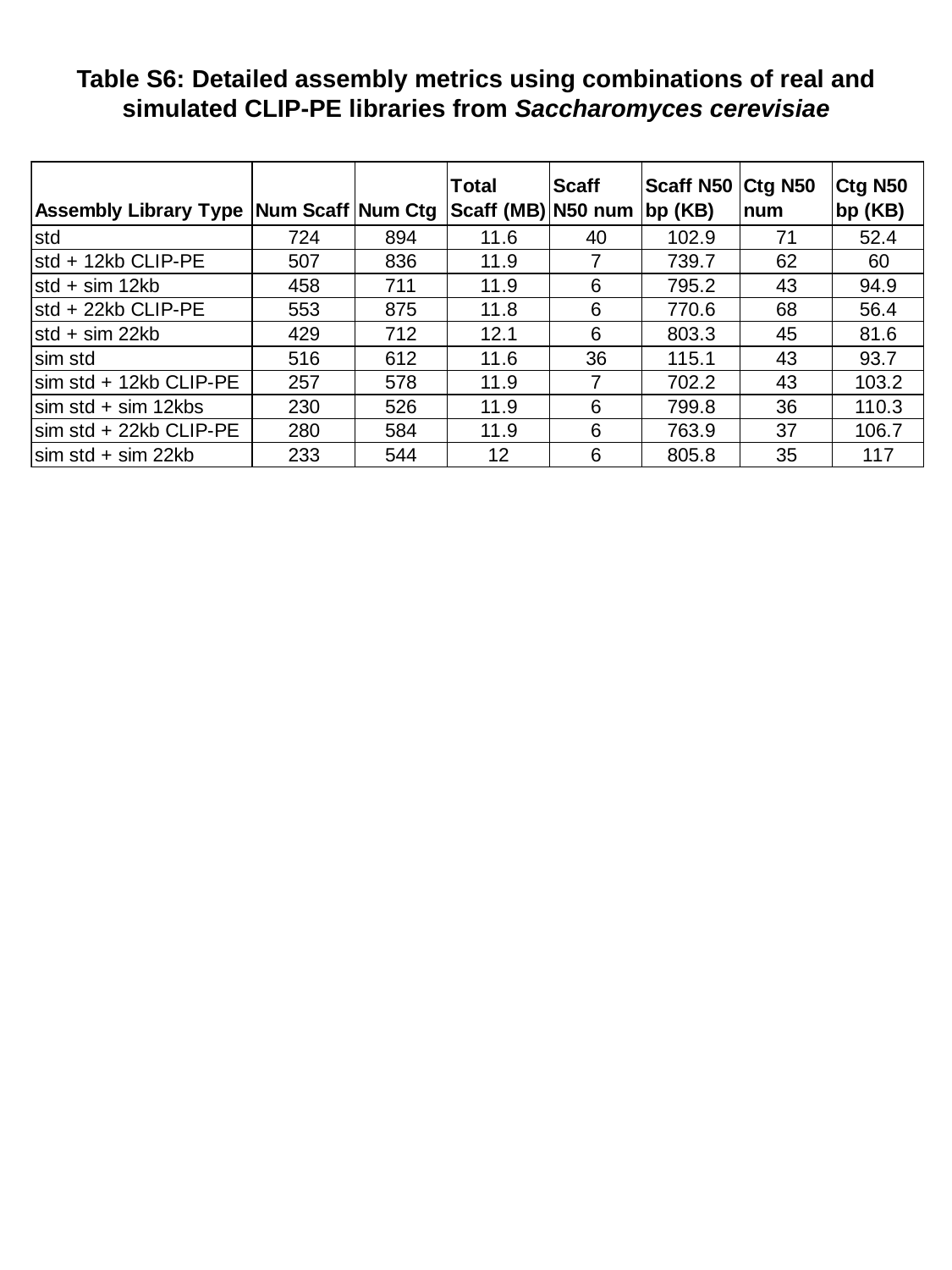

Table S6: Detailed assembly metrics using combinations of real and simulated CLIP-PE libraries from Saccharomyces cerevisiae
